# DNA Polymerase Gamma Acetylation Governs Mitochondrial Homeostasis and Vascular Cell Senescence

**DOI:** 10.7150/ijbs.122298

**Published:** 2026-02-04

**Authors:** Pengbo Wang, Liming Yu, Kexin Cao, Xiaofan Guo, Lufan Sun, Shu Zhang, Tong Zhao, Yao Yu, Mengyao Xiong, Chang Liu, Naijin Zhang, Yingxian Sun, Guozhe Sun, Liu Cao

**Affiliations:** 1 Department of Cardiology, First Hospital of China Medical University, Shenyang City, P. R. China.; 2 Health Sciences Institute, China Medical University, Shenyang City, 110122, P. R. China.; 3 State Key Laboratory of Frigid Zone Cardiovascular Disease, Department of Cardiovascular Surgery, General Hospital of Northern Theater Command, 83 Wenhua Road, Shenyang City 110016, P. R. China.; 4 Key Laboratory of Environmental Stress and Chronic Disease Control and Prevention, Ministry of Education, China Medical University, Shenyang City, P. R. China.; 5 Clinical Translational Research Center, Shengjing Hospital, Health Sciences Institute, China Medical University, Shenyang, Liaoning Province, China; 6 Innovation Center of Aging Related Disease Diagnosis and Treatment and Prevention, Jinzhou Medical University, Jinzhou, Liaoning, China.; 7 Institute of Psychiatry and Neuroscience, Henan Medical University, Xinxiang, Henan, China.

**Keywords:** DNA polymerase gamma, Acetylation, senescence, mitochondrial homeostasis, human aortic smooth muscle cells

## Abstract

DNA polymerase gamma (Polγ), the sole polymerase for mitochondrial DNA (mtDNA), emerges as a critical regulator of metabolism-associated senescence. While lysine acetylation represents a key post-translational modification (PTM) influencing mitochondrial function, its mechanistic role in Polγ-mediated vascular aging remains undefined. Through combinatorial approaches employing *in vitro* acetylation models and *POLG*^D257A/D257A^ mice, a validated model of mitochondrial dysfunction and senescence, we identify Lys 1039 (K1039) as a novel acetylation site which was dynamically regulated during aging process. Both D257A mutation-driven hyper-acetylation of Polγ K1039 reduced human aortic smooth muscle cell (HASMC) contractility, triggering pathological hyperproliferation and mitochondrial dysfunction, collectively culminating in premature cellular senescence. Pathological stimulation or genetic manipulation inducing hyperacetylation at K1039 disrupts Polγ's binding capacity with mtDNA. This molecular deficiency manifested functionally as compromised contractile performance in HASMCs and accelerated senescence phenotypes. Based on the above foundation and *POLG*^D257A/D257A^ mice model, we demonstrated that D257A mutation reduced Sirt3-Polγ complex formation constituted the pathologically relevant molecular pathway driving aberrant acetylation homeostasis and leading to the senescence. Our findings establish a previously unrecognized regulatory axis wherein Polγ acetylation status at K1039 serves as a molecular switch coordinating mtDNA homeostasis, HASMCs functionality, and senescence progression. This mechanism might explain the remarkably consistent phenotypic manifestations of Polγ-induced dysfunction across diverse tissues and aging models. This work provides fundamental insights into the epigenetic-metabolic crosstalk governing vascular aging processes, providing a unifying framework for age-related vascular pathologies.

## Introduction

Aging of HASMCs, whether through natural aging processes or disease-induced premature senescence, induces characteristic functional deterioration marked by impaired contractile capacity and compromised vascular homeostasis^1^-^3^. This cellular senescence phenomenon contributes substantially to the global burden of cardiovascular pathologies, representing a critical threat to vascular health. Senescent HASMCs demonstrate profound alterations in metabolic programming, extracellular matrix interactions, and mitochondrial bioenergetics, ultimately manifesting as dysfunctional vascular remodeling^3^-^5^. While mitochondrial functional integrity is recognized as a central determinant of cellular aging trajectories, current therapeutic strategies targeting terminal effectors like ATP synthase show limited efficacy due to their inability to address upstream regulatory mechanisms^6^. This highlights the need to identify master regulators coordinating mitochondrial homeostasis during senescence.

The *POLG*-encoded Polγ serves as the polymerase for mtDNA maintenance. Its polymerase and proofreading activity ensure stable replication of respiratory chain components essential for oxidative phosphorylation^4^. Previous studies confirmed Polγ deficiency triggered a cascade of mitochondrial crises including apoptotic activation, oxidative stress amplification, and pathological vascular calcification whose phenomena particularly exacerbated under metabolic stress conditions^7^-^9^. Emerging evidence suggests Polγ may function as a nodal regulator capable of compensating for diverse mitochondrial defects, while most studies predominantly focused on its expression dysregulation in disease 10. Interestingly, we noted inconsistent expression patterns, even within same disease classifications, which provided the likelihood of alternative Polγ activation modalities, such as allosteric or PTM regulation. Protein acetylation modification, a dynamic lysine modification implicated in cardiovascular pathophysiology and aging mechanisms, represents a plausible regulatory pathway given the prevalence of acetylated mitochondrial proteome components^11^-^14^. However, whether the activity and function of Polγ are regulated through acetylation remains incompletely defined.

This work provided the first direct evidence for Polγ in PTM, uncovering a previously unrecognized regulatory mechanism through acetylation that governed its functional significance, which mitochondrial genome stability was actively regulated by acetylation-dependent signaling. Our study identifies K1039 as a novel acetylation site modulating Polγ activity in vascular aging. Using the *POLG*^D257A/D257A^ mice model of premature senescence with established mitochondrial dysfunction^15^, ^16^, we demonstrated that acetylation at K1039 impairs Polγ's DNA-binding capacity, thereby reducing mitochondrial homeostasis and cellular function. We established Polγ acetylation as a pivotal regulatory switch in vascular aging, providing a unifying mechanism connecting mitochondrial genomic instability to cellular senescence. The identification of this acetylation-dependent regulatory axis offered novel therapeutic opportunities for targeting age-related cardiovascular pathologies through precision modulation of mitochondrial homeostasis.

## Methods

### Antibodies, reagents and commercial kits

Acetylated-lysine, Cell Signaling Technology (USA) Cat #9441; Citrate Synthase, Santa Cruz (USA) Cat #sc-6246; COX IV, Wanleibio (China) Cat #WL01418; Flag-tag, Absmart (China) Cat #M20008; Flag-tag, GNI (Japan) Cat #GNI4110-FG-M; GAPDH, Proteintech (China) Cat #60004-1-Ig; GCN5, Proteintech (China) Cat #66575-1-Ig; HRP goat anti-mouse IgG, ABclonal Biotech Cat #AS003; HRP goat anti-rabbit IgG, ABclonal Biotech Cat #AS014; L-type Ca^2+^ CP, Santa Cruz (USA) Cat #sc-515679; Myc-tag, Cell Signaling Technology (USA) Cat #2276; p16, Proteintech (China) Cat #10883-1-AP; p16, Wanleibio (China) Cat #WL02203; p21, Santa Cruz (USA) Cat #sc-6246; PKM2, Cell Signaling Technology (USA) Cat #4053S; PKM2-phosphorylation, Cell Signaling Technology (USA) Cat #3827S; Polγ, Abcam (USA) Cat # ab128899; Polγ, Abcam (USA) Cat # ab97661; Polγ, Cell Signaling Technology (USA) Cat #13609; Polγ, Santa Cruz (USA) Cat #sc-390634; Sirt3, Santa Cruz (USA) Cat #sc-6246; TRPC6, Santa Cruz (USA) Cat #sc-515837; Tubulin, Proteintech (China) Cat #11224-1-AP.

FBS, Clark (Australia) Cat #FB15015; High-glucose DMEM, Biological Industries (Israel) Cat #01-052-1A; High-fat Diet Feed, Medicience (China) Cat #MD12033; HiGene, Applygen (China) Cat #C1506; Lipofectamine 2000, Thermo Scientific (USA) Cat #11668019; NAM, MedChemExpress (USA) Cat #HY-101407; PageRuler™ Multicolor High Range Protein Ladder, Thermo Scientific (USA) Cat #26625; PageRuler™ Prestained Protein Ladder, Thermo Scientific (USA) Cat #26616; Protein A/G Magnetic Beads, Selleckchem (USA) Cat #B23202; Sirt3 activator, MedChemExpress (USA) Cat #HY-139742; Sirt3 inhibitor, MedChemExpress (USA) Cat #HY-108331; Streptozotocin, MedChemExpress (USA) Cat #HY-13753; TSA, MedChemExpress (USA) Cat #HY-15144; Type I Collagen Solution, Solarbio (China) Cat #C8062; Vit D_3_ solution, Solarbio (China) Cat #V8070; Cell Counting Kit-8, Beyotime (China) Cat #C0037; Chromatin IP Kit, Merck (USA) Cat #17-10086; JC-10 Assay Kit, Solarbio (China) Cat #CA1310; Mitochondrial Extraction Kit, Solarbio (China) Cat # SM0020; SA-Β-Gal Staining Kit, Beyotime (China) Cat #C0602; SimpleChIP® Enzymatic Chromatin IP Kit (Magnetic Beads), Cell Signaling Technology (USA) Cat #9003.

### Mouse models

The *POLG*^D257A/D257A^ mice were generously provided by Professor Nils-Göran Larsson. Wild-type (WT) littermates *POLG*^WT/WT^ mice and C57/BL6 WT mice served as controls. All mice were maintained under specific pathogen-free conditions at 21-23°C with ad libitum access to standard chow and distilled water. Animal protocols were approved by the Institutional Animal Care and Use Committee (IACUC) of China Medical University and conducted in compliance with NIH guidelines (Publication No. 85-23, revised 1985).

For vascular calcification induction, mice received subcutaneous injections of vitamin D3 (8×10⁵ IU/kg in corn oil) daily for 3 days and were euthanized on day 12^7^. To model diabetic vascular injury, mice were fasted for 5 days, administered streptozotocin (40 mg/kg, intraperitoneal), and maintained on a high-fat diet for 6 weeks^17^.

### Cell lines and cell culture

HASMCs and HEK293T cells were generously provided by Professor Liu Cao. Sh-Polγ HASMC lines were generated as previously described^7^. Cells were cultured in high-glucose Dulbecco's modified Eagle's medium (DMEM) supplemented with 10% FBS, 100 U/mL penicillin, and 100 μg/mL streptomycin at 37°C under 5% CO₂.

### Mouse embryonic fibroblasts (MEFs) and primary HASMC isolation

MEFs were isolated from E12.5 fetal skin tissues. Tissues were digested with EDTA-free trypsin (0.25%, w/v), and cells were cultured in DMEM containing 15% FBS at 37°C with 5% CO₂. Primary HASMCs were isolated from and 8-week-old murine thoracic aortae. Following microdissection to remove adventitial connective tissue and the tunica intima, the aortic tunica media layer was isolated, mechanically minced, and subjected to enzymatic digestion with EDTA-free trypsin (0.25%, w/v) to dissociate smooth muscle cells, and cells were cultured in DMEM containing 15% FBS at 37°C with 5% CO₂ for downstream functional analyses.

### Mass spectrometry acetylation modification omics

Samples preparations were following standard procedures for acetylation modification omics analysis. Acetylated peptides were enriched using iTRAQ labeling and anti-acetyl-lysine antibody-conjugated agarose beads. After 1-hour incubation at 4°C, peptides were eluted and analyzed by LC-MS/MS. Spectral counting quantified acetylation levels.

### Western blot (WB) and co-immunoprecipitation (Co-IP) analysis

Cells or tissues were lysed on ice (30 min) and vortexed every 10 min, centrifuged (13,000 ×g, 20 min), and quantified. Lysates (30-50 μg) were resolved on 8% or 12% SDS-PAGE gels, transferred to PVDF membranes (80 V, 150 min), and blocked with 5% non-fat milk or BSA blocking solution. Primary antibodies (1:200-1,000 dilution) were incubated overnight at 4°C, followed by HRP-conjugated secondary antibodies (1 h, RT). Chemiluminescent signals were quantified using ImageJ v1.46 (National Institutes of Health, USA https://imagej.nih.gov/ij/), normalized to Tubulin or GAPDH. For Co-IP, lysates were incubated with antibodies (3 h, 4°C), followed by protein A/G beads (12 h, 4°C). Precipitates were analyzed by WB.

### Chromatin immunoprecipitation (Ch-IP) analysis

Ch-IP assay was performed according to the manufacturer's protocols. In brief, prepared samples were cross-linked with 1% formaldehyde at room temperature for 10 minutes and chromatin was lysed into fragments with sonication following addition of Glycine for 5 minutes. Then the samples were incubated with complex of primary antibodies and protein A/G beads overnight at 4℃. On the next day, immunoselected DNA fragments were purified after the beads-antibody-DNA fragment complex was collected and reverse cross-linked. Finally, DNA fragments were used for qPCR experiments. Forward Primer for Ch-IP, CTGAGCTCACCATAGTCTAATAG; Reverse Primer for Ch-IP, GATGTTTGGATGTAAAGTGAAAT; The results of Ch-IP analysis were exhibited as percentage of input chromatin and were derived from a single experiment that is representative of at least three independent experiments.

### Subcellular component isolation

Mitochondria were isolated from freshly collected cells using a mitochondrial extraction kit. First, the cells were ground with a glass grinder and then broken with lysis buffer. The samples were centrifuged at 1000 g for 5 min at 4 ° C, twice, and then centrifuged at 12,000 g for 10 min. The supernatant was collected as cytoplasmic protein. Then wash the pellets with wash buffer and centrifuged at 12,000 g for 10 min at 4 ° C. The precipitate was collected as mitochondria.

### Co-localization analysis

The cell slides were placed into 24-well plates with clean forceps, and then the cells were cultured. When the cells grew to 70-80% density, the cell culture-medium was discarded and washed with PBS. Precooled methanol solution was used for fixation at room temperature for 3min, followed with three times washing with PBS. Then the slides were drilled with 0.25%Triton-100 for 15min at room temperature and washed with PBS at the end. After that, the cells were blocked with 5% BSA solution at room temperature for 1 hour, and 300ul of primary antibody in 5% BSA solution was added. The cells were incubated overnight at 4 °C. The next day the plates were washed with PBS, and then were incubated with fluorescent secondary antibodies at room temperature for 1h in the dark place. After the incubation, the cells were washed with PBS again and the DAPI solution was used to stain the nuclei. Then wash the cells again with PBS, and the slides were placed on microscope slides and sealed with antifade mounting medium. Fluorescence co-localization was observed and photographed using a confocal microscope at 40x magnification. The mean fluorescence intensity was calculated using Image J for comparison between groups.

### Plasmid construction and transfection

The methods of constructing Polγ-WT plasmid and Polγ-D257A plasmid has been described in previous articles^7^. In this study, the PCR technology was used to construct point mutation plasmids. We made exogenous gene expression by transfection^18^. Transfection was performed when the cells had grown to 60% confluence. The transfection mixture containing plasmid (10μg DNA for each plasmid in a 10cm cell culture dish, the vector plasmid was used as control group), transfection reagent Lipofectamine 2000 or HiGene and PBS was placed at 37 °C for 15min after thorough mixing, and then it was added to the cell culture medium. The medium was changed after 6 hours, and the cells were collected after 48 hours for the following analysis experiments.

### Cell viability assay

We used Cell Counting Kit-8 (CCK-8) to evaluate the cell viability. The treated cells were inoculated into 96-well plates with 8000 cells for each well and incubated in incubator. On the next day, 10 µl of CCK-8 solution was added to each well and incubated for 90 min in the incubator. Detect the absorbance at 450 nm to evaluate the proliferative ability and activity of the cells.

### Colony forming assay

The digested cells were counted and cultured into 6cm cell culture dishes with 3000 cells per dish. The cells were cultured with DMEM for 7 days, and the medium was changed every two days. Staining and photography were performed on day 0/3/7, respectively. Before staining, the medium was dropped, the cells were fixed with paraformaldehyde at room temperature for 15min, and then washed it with PBS. 0.1% crystal violet staining solution was used for staining at room temperature for 15min. After staining, the non-specific staining was washed away with PBS. Taking photograph under the microscope, a colony was defined as more than 50 cells. The colonies were counted by Image J, and colony formation rate was calculated.

### SA-β-Gal staining

We performed SA-β-Gal staining to observe the senescent level of cells. After discarding the culture medium and washing the cell surface with PBS solution, we fixed the cells with paraformaldehyde at room temperature for 15 minutes. After fixation, the cell fixative was aspirated and the cells were washed with PBS three times, each time for 3 minutes. The prepared staining solution was added to the culture dish and the culture dish was sealed and then placed in a 37ºC incubator without CO_2_ overnight. On the next day, we discarded the staining solution and added 2 milliliters of PBS. The staining was observed under a microscope and photographs were taken. The ImageJ was used to count the proportion of color-positive cells in the field as a measure of the degree of aging.

### Collagen gel contraction assays

We conducted collagen gel contraction experiment to observe the contraction ability of HASMC. The cells were digested, counted and then diluted into a single-cell suspension. We mixed 300 μl of cell suspension, 100 μl of type I collagen solution, 94 μl of DMEM, and 6 μl of NaOH solution carefully on ice. The prepared cell gel was seeded in a 24-well plate and placed in a 37°C incubator for 30 minutes. After gel formation, 500 μl of culture medium was added to each well and the gel was gently detached from the well walls. Then the plate was incubated in a 37°C incubator. The photographs were taken using a gel imaging system at 0 hours and 12 hours after gel formation, and the surface area of the collagen gel was measured using ImageJ software. The percentage of gel surface area contraction (%) was calculated using the formula: [(0h area - 12h area) / 0h area] × 100%.

### Mitochondrial membrane potential (MMP) determination

We used JC-10 assay kit to determine the MMP and further evaluate the mitochondrial function. We digested the cells and resuspended them into a single-cell suspension using culture medium. The prepared JC-10 staining solution was added and mixed thoroughly. Then the cells were incubated in a light-protected cell culture incubator at 37°C for 20 minutes. After incubation, the cells were centrifuged at room temperature. Then we aspirated supernatant and washed cells twice with PBS solution, followed by adding 2 mL of culture medium to resuspend into a single-cell suspension, and the MMP was analyzed using flow cytometry with FITC and PE channels. The ratio was calculated to evaluate the MMP and mitochondrial function.

### Molecular modeling

We used SWISS-MODEL (https://www.swissmodel.expasy.org) to model the three-dimensional (3D) structural coordinates of Polγ to provide molecular structure evidence for our hypothesis which D257A mutation might bring alteration in acetylation modification and protein binding levels. The amino acid sequence of Polγ was obtained from the NCBI database and the sequence was submitted to the SWISS-MODEL web server to generate the models based on query sequence coverage and identity. We used the Global Model Quality Estimation (GMQE) and Qualitative Model Energy Analysis (QMEAN) values accessing system to guarantee the reliability of 3D structure, and we eventually selected the model with the highest GMQE value and the QMEAN value below 4.0.

### AlphaFold-structure prediction

To predict the structural consequences of lysine acetylation, we employed AlphaFold3 for protein structure modeling. The WT Polγ sequence was submitted to AlphaFold3 to generate a baseline structural model. To simulate acetylated lysine residues, we introduced acetyl-lysine analogs at specified positions (K1039) by modifying the residue type in the input sequence using a structural parameter set compatible with the CHARMM36 force field. Five models were generated for each variant, and the model with the highest predicted confidence score was selected for further analysis. Structural comparisons between wild-type and acetylated forms were performed by aligning the predicted models in PyMOL (version 2.5.4).

### Molecular docking

The protein structures of Polγ (WT and D257A) and Sirt3 were obtained from AlphaFold-predicted models. These structures were downloaded and converted into the GROMACS-specific format (.gro) using GROMACS software (version 2022.5). During this conversion process, missing atoms were repaired, and defects in terminal residues were ignored to ensure the completeness of the structure files and the accuracy of subsequent simulations. To simulate the dynamic behavior of Polγ (WT and D257A) and Sirt3 under physiological conditions, a solvated system was constructed using GROMACS modules, including editconf, solvate, and genion. The proteins were placed in a water box, and counter-ions were added to ensure system neutrality. The system underwent two stages of equilibration: NVT (constant volume constant temperature) to stabilize the temperature while maintaining a constant volume, and NPT (constant pressure constant temperature) to stabilize the pressure and mimic physiological conditions. Molecular docking was performed using ClusPro 2.0 (https://cluspro.bu.edu/), where docking simulations of Polγ (WT and D257A) and Sirt3 proteins with small molecules (both modified and unmodified) were conducted. The docking pose with the lowest binding energy, indicating the highest binding affinity, was selected as the final result.

### Quantification and statistical analysis

The data are presented as mean ± SD. The F-test (two groups) or the Brown-Forsythe test (three or more groups) was used to assess the homogeneity of variance. The Shapiro-Wilk test was used to assess the normality of the data. Student's t-test and Welch's t-test were used for equal variance and unequal variance, respectively (two groups). Two-way analysis of variance with the Bonferroni multiple-comparisons test was used when two conditions were considered between the groups. The p values were adjusted for multiple comparisons where appropriate. GraphPad Prism 8.0 software, SPSS version 22.0 (IBM Corp., Armonk, NY, USA https://www.ibm.com/spss) and R software (version 4.0.5 http://www.R-project.org) were used for all statistical analyses, with a P value less than 0.05 indicating statistical significance.

## Results

### Polγ could undergo acetylation and is augmented in senescent HASMC

**Figure 1:**
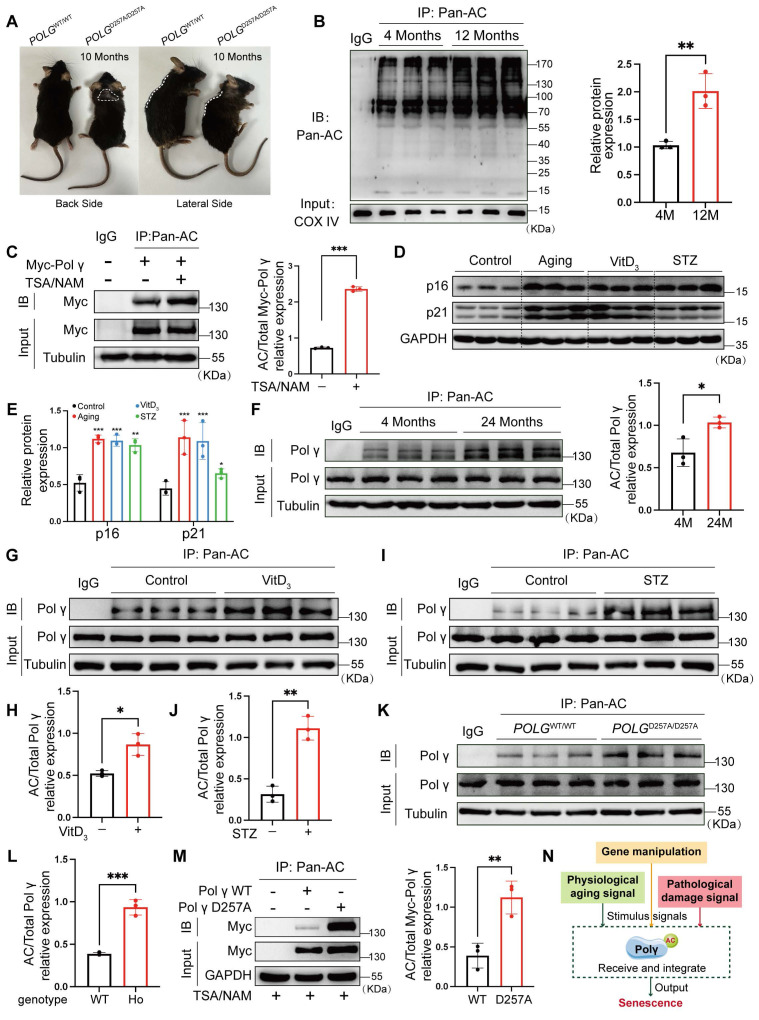
** Polγ acetylation is augmented during senescence and regulated by D257A mutation.** (A) The difference of morphological characteristics between *POLG*^WT/WT^ mice and *POLG*^D257A/D257A^ mice; (B) The global acetylation of mitochondrial protein among different age in mice aorta by Co-IP assay, the right panel is the quantification analysis. COX IV was used as a loading control, **p < .01; (C) Co-IP analysis and quantification (right panel) of relative acetylation level of Polγ in HASMCs which treated with TSA/NAM for 24 hours. The expression of Myc in input was used as a loading control. ***p < .001; (D, E) The molecular marker expression of senescence in aging model, vascular calcification model and diabetes model by WB assay and its quantification analysis. The expression of GAPDH was used as a loading control. ***p < .001. **p < .01 *p < .05; (F-J) The acetylation of Polγ in different models by Co-IP assay and their quantification analysis. (F) for 4 months and 12 months mice aorta, (G, H) for control and VitD3 induced vascular calcification model and (I, J) for control and STZ induced vascular diabetes model. The expression of Polγ in input was used as a loading control. ***p < .001, *p < .05; (K, L) The acetylation of Polγ in aorta of *POLG*^WT/WT^ mice and *POLG*^D257A/D257A^ mice by Co-IP assay and its quantification analysis. The expression of Polγ in input was used as a loading control. **p < .01; (M) Transfect the HASMCs with Vector plasmid, Polγ-WT plasmid and Polγ-D257A plasmid for 48 hours respectively, and detect their acetylation level by Co-IP assay. The right panel is the quantification analysis. The expression of Myc in input was used as a loading control. **p < .01; (N) Schematic diagram. All data were expressed as the mean ± SD of triplicate experiments. In each independent experiment, the sample size for each group of experiments is 3.

Previous studies established that the D257A mutation in Polγ induces premature aging phenotypes in mice, including shortened lifespan, spinal deformities, and alopecia (Figure 1A). Given the regulatory role of acetylation in protein function, we observed a hyper-acetylated state in mitochondrial proteins of aged mice (Figure 1B). Co-immunoprecipitation (Co-IP) assays confirmed Polγ acetylation, which was further enhanced by deacetylase inhibitors TSA/NAM (Figure 1C). We also performed physiological aging model, vascular calcification model and diabetes model, and we observed the expression of senescent marker p16 and p21 were elevated in all three models which indicated these common pathological models were all accompanied with senescence process (Figure 1D, E). Furthermore, the respectively Co-IP experiment indicated the acetylation of Polγ were all up-regulated in these senescent models, suggesting the acetylation of Polγ could be regulated and involved into different aging related processes (Figure 1F-J). Based on the above results, we compared *POLG*^D257A/D257A^ mice with *POLG*^WT/WT^ mice and observed Polγ-D257A exhibited a higher acetylation level which was also confirmed *in vitro* (Figure 1K-M).

These results demonstrated Polγ could undergo acetylation modification which could be dynamically regulated during senescence and contributed to D257A mutation-induced aging. Based on these findings, we proposed that Polγ integrated diverse senescence-inducing signals, whether arising from genetic deficiency, physiological processes or pathological conditions, and transduced senescence-related effector signals through modulation of its acetylation status (Figure 1N).

### D257A mutation elevated acetylation level, reducing HASMCs function and disrupting the mitochondrial homeostasis

**Figure 2:**
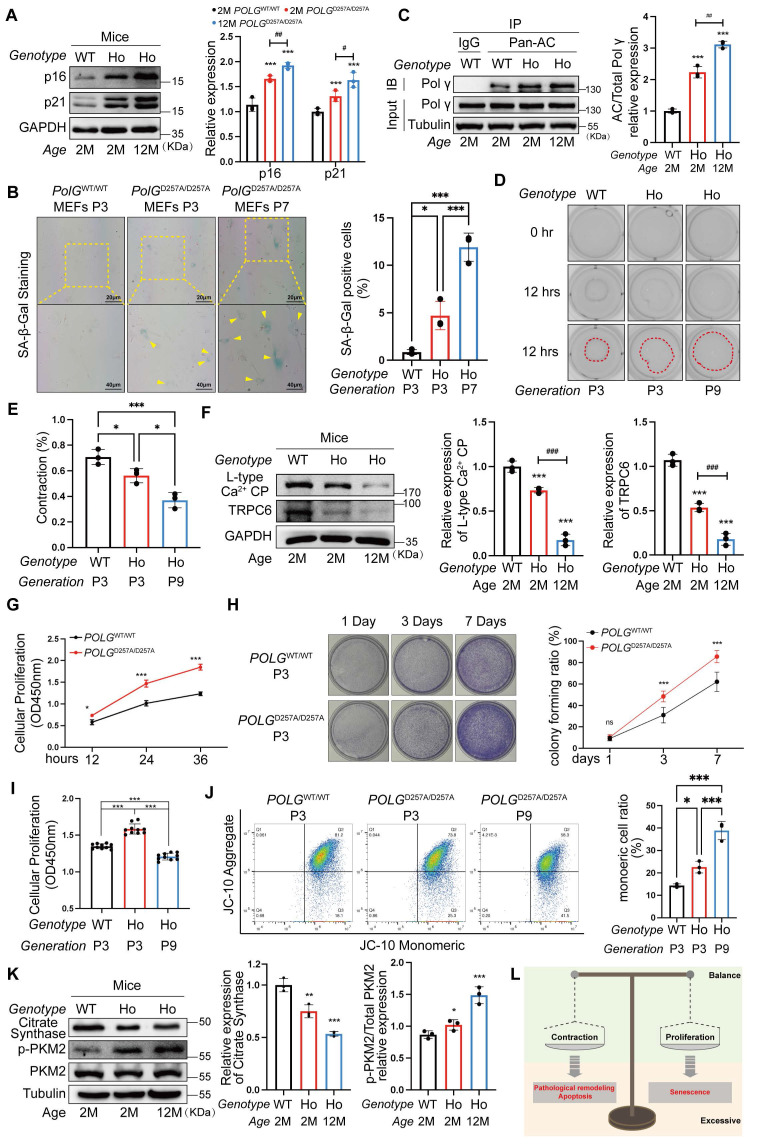
** D257A mutation exacerbates senescence via dysregulated HASMC function and mitochondrial dysfunction.** (A) The different expression of senescence marker in the aorta of *POLG*^WT/WT^ mice and *POLG*^D257A/D257A^ mice by WB assay, the right panel is the quantification analysis. WT is for* POLG*^WT/WT^ mice and HO is for *POLG*^D257A/D257A^ mice. The expression of GAPDH was used as a loading control. ***p < .001. ^##^p < .01, ^#^p < .05; (B) SA-β-Gal staining for MEFs with different genotype and passage, the rate of positive staining cells was counted to analysis. ***p < .001, *p < .05; (C) Co-IP analysis and quantification analysis of relative acetylation level of Polγ in aorta of different genotype and age. ***p < .001. ^##^p < .01; (D-E) The collagen gel contraction experiment of primary HASMCs with different genotype and passage. The percentage of gel surface area contraction (%) was calculated using the formula: [(0h area - 12h area) / 0h area] × 100%. ***p < .001, *p < .05; (F) The expression of various Ca^2+^ channel related proteins in aorta of *POLG*^WT/WT^ mice and *POLG*^D257A/D257A^ mice by WB assay, the median and right panels are quantification analysis. The expression of GAPDH was used as a loading control. ***p < .001, ^###^p < .001; (G) The cell viability experiment of primary HASMC of *POLG*^WT/WT^ mice and *POLG*^D257A/D257A^ mice by CCK-8 kit assay in 12 hours, 24 hours and 36 hours. ***p < .001, *p < .05; (H) The colony forming experiment of primary HASMC of *POLG*^WT/WT^ mice and *POLG*^D257A/D257A^ mice, the cells were cultured for 7 days, staining and counting the colony numbers in 1day, 3days and 7days. Counting the colony numbers to analysis. ***p < .001, *p < .05, ns p>0.05; (I) The cell viability experiment of primary HASMC of *POLG*^WT/WT^ mice and *POLG*^D257A/D257A^ mice with different passage by CCK-8 kit assay in 12 hours, 24 hours and 36 hours. ***p < .001; (J) The JC-10 measurement of MMP of the primary HASMC of different genotype and passage by flow cytometry. Compare the difference of monomeric cells ratio (green) in every subgroup. ***p < .001, *p < .05; (K) The expression of metabolic proteins (citrate synthase, p-PKM2, PKM2) in aorta of different genotype and age. The expression of Tubulin was used as a loading control for citrate synthase, and PKM2 was used as a loading control for p-PKM2. The median and right panels are quantification analysis, ***p < .001, **p < .01, *p < .05; (K) Schematic diagram. All data were expressed as the mean ± SD of triplicate experiments. In each independent experiment, the sample size for each group of experiments is 3.

To reveal the role of Polγ D257A mutation in HASMC function and senescence process, we compared the difference among *POLG*^WT/WT^ mice, *POLG*^D257A/D257A^ (homozygote mice, HO) mice and senescent *POLG*^D257A/D257A^ mice. In *POLG*ᴰ²⁵⁷ᴬ/ᴰ²⁵⁷ᴬ mice, p16/p21 expression were markedly elevated, worsening with age (Figure 2A). In the proliferative aging model, we performed the SA-β-gal staining and indicated the *POLG*^D257A/D257A^ MEFs of P3 has already exhibited senescence and further aggravated in P7 (Figure 2B). Concurrently, Polγ acetylation increased progressively in* POLG*^D257A/D257A^ mice and further aggravated with age (Figure 2C). Functional assays revealed impaired contractility in Polγ-mutant HASMCs which even lost contraction ability in senescent HASMCs (Figure 2D, E), linked to reduced L-type Ca²⁺ channel (LCP) and TRPC6 expression which were important influx Ca^2+^ marker and essential for maintain the contractive ability (Figure 2F). Paradoxically, *POLG*ᴰ²⁵⁷ᴬ/ᴰ²⁵⁷ᴬ HASMCs exhibited hyperproliferation via Cell Counting Kit-8 (CCK8) assay and colony formation assays no matter they were young or senescent suggesting a abnormal high proliferation rate (Figure 2G, H). And we noted the D257A mutation attenuates senescence-associated decline in proliferative capacity, as evidenced by the robust proliferative capability of Passage 9 (P9) D257A HASMCs which remained comparable to that of P3 WT HASMCs (Figure 2I). Finally, we also examined mitochondrial function and found that the D257A mutation diminished mitochondrial membrane potential (MMP) levels, which further worsened with aging (Figure 2J). Mitochondrial dysfunction was also evident through suppressed citrate synthase (CS), and elevated pyruvate kinase isozyme type M2 (PKM2) phosphorylation in *POLG*ᴰ²⁵⁷ᴬ/ᴰ²⁵⁷ᴬ HASMCs and even more obvious in senescent cells, indicating a metabolic shift toward glycolysis (Figure 2K).

Herein, we observed D257A mutation driven senescence via disrupted mitochondrial homeostasis and aberrant HASMC function which were diminished contraction ability and hyperproliferation. HASMCs maintained a dynamic balance between contractile phenotype and proliferative phenotype. This “phenotypic equilibrium” shifted toward either phenotype in response to microenvironmental alteration. Pathologically, however, HASMCs would develop hyper-contractile phenotypes associated with apoptosis and pathological vascular remodeling, or undergo hyper-proliferation-driven premature senescence (Figure 2L). Critically, D257A mutation mediated Polγ acetylation might be the effect process which disrupted phenotypic balance of HASMCs which further leading to senescence.

### The acetylation modification of Polγ is mediated by GCN5/Sirt3

**Figure 3:**
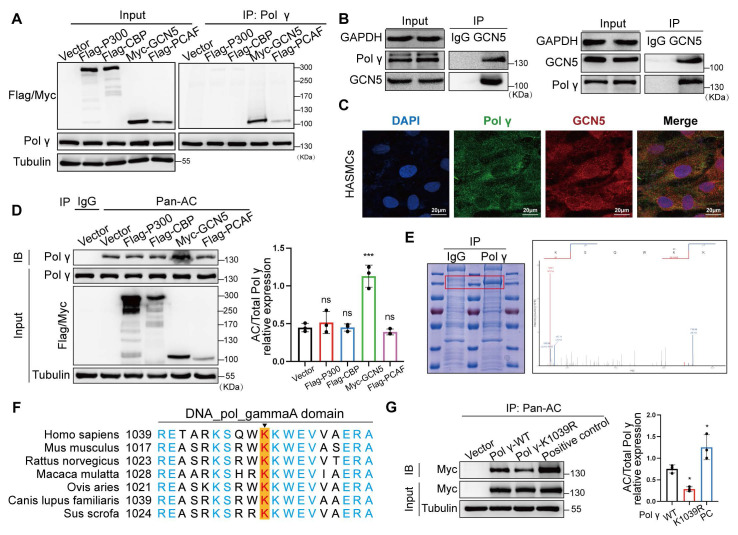
** GCN5 is the acetyltransferase for Polγ acetylation at K1039.** (A) The Co-IP assay to screen the interaction between Polγ and acetyltransferases. (B) The Co-IP assay to confirm the interaction between Polγ and GCN5. (C) The interaction between Polγ and GCN5 by confocal assay. (D) The effect of various acetyltransferases in acetylating Polγ. Right panel is the quantification analysis. The expression of Polγ in input was used as a loading control. ***p < .001, ns p>0.05; (E) Enrich endogenous Polγ and conduct mass spectrometry acetylation modification omics to confirm the acetylated site of Polγ. (F) The conserved analysis of DNA sequence around K1039 site of Polγ in different species. (G) The CO-IP experiment and quantification of relative acetylation level of different types of Polγ. The positive control was additionally treated with TSA+NAM to inhibit deacetylation on the basis of Polγ-WT transfection. The expression of Myc in input was used as a loading control. *p < .05. Data were expressed as the mean ± SD of triplicate experiments. All data were expressed as the mean ± SD of triplicate experiments. In each independent experiment, the sample size for each group of experiments is 3.

To delineate the molecular pathways governing Polγ acetylation, we systematically screened acetyltransferases and deacetylases regulating this post-translational modification. After overexpressing common acetyltransferases to HASMCs, acetyltransferase screening identified GCN5 as the primary enzyme mediating Polγ acetylation, validated by endogenous Co-IP and colocalization assay (Figure 3A-C). Meanwhile, we observed GCN5 significantly improved the acetylation level of Polγ (Figure 3D). The above results suggested that GCN5 was the acetyltransferase of Polγ. Next, we enriched Polγ protein and conducted mass spectrometry acetylation modification omics, the results pinpointed K1039 site which was highly conserved among several species might undergo acetylation (Figure 3E-F). Thus, we confirmed whether K1039 was the acetylation site of Polγ by acetylation deficiency mutation. By transfecting Polγ-WT plasmid and Polγ acetylation-deficiency plasmid (Polγ-K1039R), we observed that Polγ-WT had appropriate basal level acetylation modification, K1039R mutation reduced acetylation modification, these results confirmed this site's role in modulating acetylation levels (Figure 3G).

**Figure 4:**
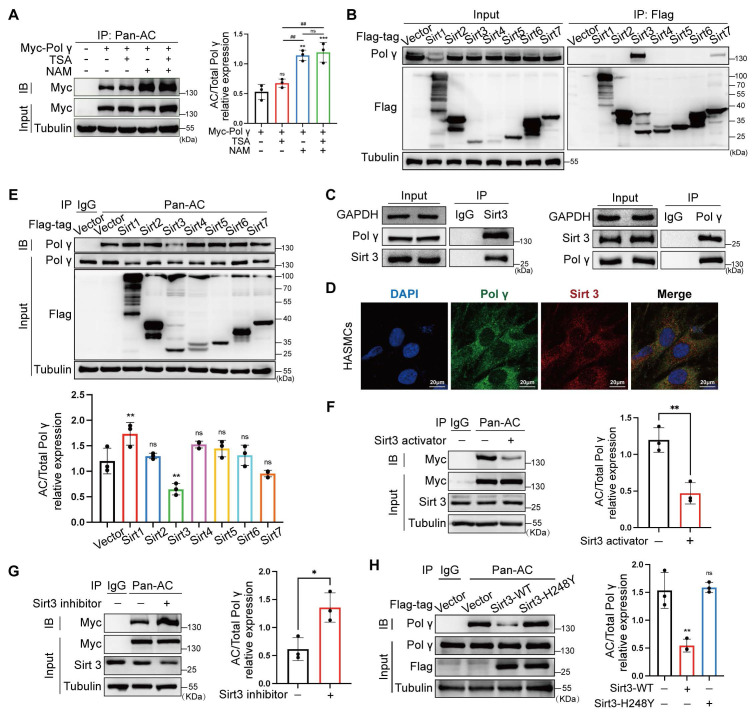
** Sirt3 serves as deacetylase in Polγ acetylation.** (A) The CO-IP assay and quantification analysis of relative acetylation level of Polγ. The HASMC was treated with TSA/NAM for 24 hours. The expression of Myc in input was used as a loading control. ***p < .001, **p < .01, ^##^p < .01, ns p>0.05; (B) Screen the interaction between Polγ and various deacetylases by Co-IP assay. (C) Confirm the interaction between Polγ and Sirt3 by Co-IP assay; (D) Confirm the interaction between Polγ and Sirt3 by confocal assay; (E) Compare the capacity of various deacetylases in deacetylating Polγ. The bottom is the quantification analysis. The expression of Polγ in input was used as a loading control. **p < .01, ns p>0.05; (F-G) Detect the acetylation level of Polγ with treatment of Sirt3 activator (F) or Sirt3 inhibitor (G) in HASMCs by Co-IP assay. The right panel is the relative quantification analysis. **p < .01, *p < .05; (H) The Co-IP experiment and quantification of relative acetylation level of Polγ, the HASMC was transfected with Sirt3 and deacetylase activity deficiency Sirt3 (H248Y). The expression of Myc in input was used as a loading control. *p < .05. All data were expressed as the mean ± SD of triplicate experiments. In each independent experiment, the sample size for each group of experiments is 3.

As nicotinamide (NAM, a Sirtuins inhibitor) increased Polγ acetylation while histone deacetylase (HDAC) family inhibitors trichostatin A (TSA) had no effect, we focused on the Sirtuins family (Figure 4A). The Co-IP experiment showed Sirt3 had strongest binding with Polγ, which was further confirmed by endogenous Co-IP and colocalization experiments (Figure 4B-D). By acetylation modification experiments, Sirt3 emerged as the dominant deacetylase counteracting the acetylation of Polγ (Figure 4E), with pharmacological agents confirming its regulatory role. Sirt3 activator reduced Polγ acetylation and Sirt3 inhibitor elevated Polγ acetylation (Figure 4F, G). Sirt3 had strongest deacetylation effect than other Sirtuins family deacetylases in mitochondrial and we found Sirt3 deacetylase activity deficiency mutation (H248Y) lost the effect in down-regulating Polγ acetylation (Figure 4H).

These findings establish a GCN5/Sirt3-K1039 axis as the central molecular pathway modulating Polγ acetylation.

### D257A mutation reduced the binding capacity of Polγ with mtDNA by elevating the K1039 acetylation

**Figure 5:**
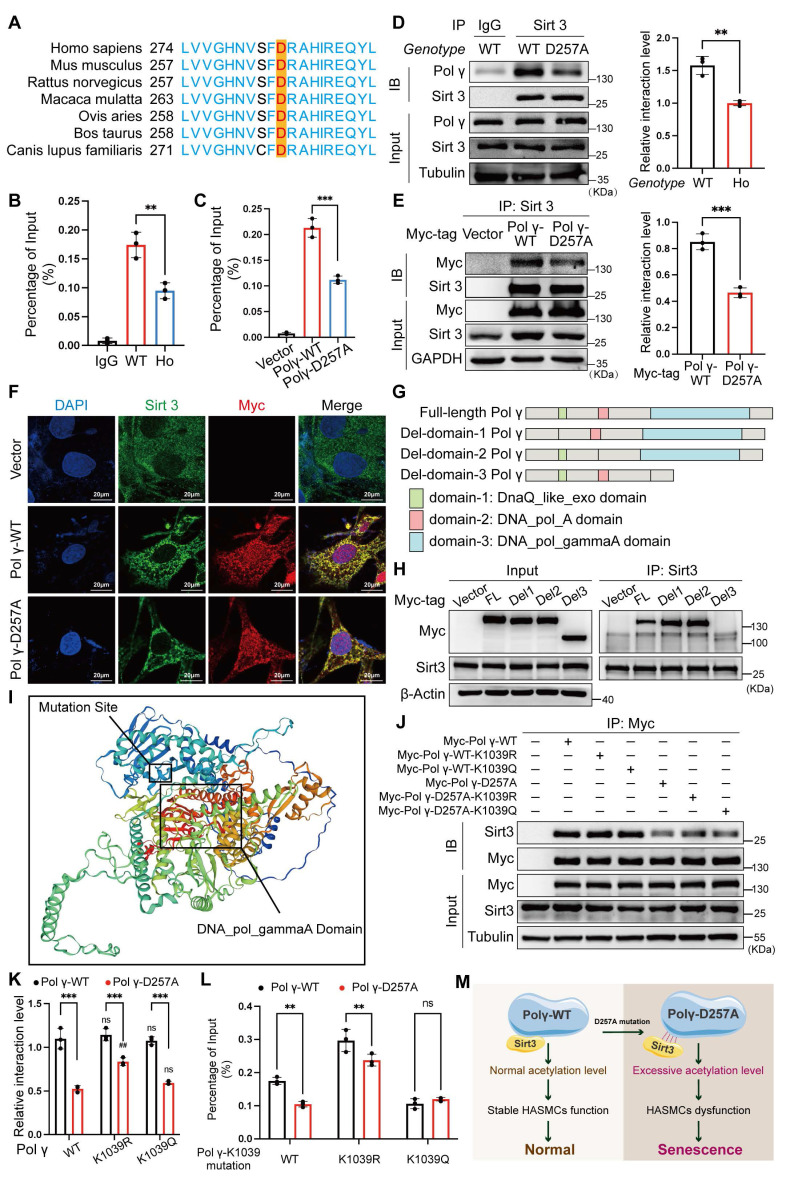
** D257A mutation impairs poly polymerase activity via enhanced K1039 acetylation.** (A) The conserved analysis of DNA sequence around D257 site of Polγ in different species; (B-C) The Ch-IP experiments to evaluate the binding degree between Polγ and mtDNA *in vivo* (B) and *in vitro* (C). WT for *POLG*^WT/WT^ mice and HO for *POLG*^D257A/D257A^ mice. ***p < .001, **p < .01; (D-E) Compare the interaction between Sirt3 and different types of Polγ *in vivo* (D) and *in vitro* (E) by Co-IP assay. The right panel is quantification analysis. ***p < .001, **p < .01; (F) Compare the interaction between Sirt3 and different types of Polγ by confocal; (G) Schematic diagram of domain-deletion plasmid of Polγ; (H) Clarify the interaction domain of Polγ with Sirt3 by Co-IP assay; (I) The predictive 3D structure of Polγ by SWISS-MODEL; (J-K) The Co-IP experiment and quantification analysis to evaluate the interaction degree between Sirt3 and different types of Polγ. ***p < .001, ^##^p < .01, ns p>0.05; (L) Evaluate the binding degree between different types of Polγ and mtDNA by Ch-IP assay. **p < .01, ns p>0.05; (M) Schematic diagram. All data were expressed as the mean ± SD of triplicate experiments. In each independent experiment, the sample size for each group of experiments is 3.

The D257 residue is critical for Polγ function, which is evolutionarily conserved across species (Figure 5A). By *in vivo* and *in vitro* chromatin immunoprecipitation (Ch-IP) assays, we found that the D257A mutation significantly weakened Polγ's binding affinity to mtDNA (Figure 5B, C). Co-IP and co-localization assays further demonstrated that this mutation disrupted the interaction between Polγ and Sirt3 (Figure 5D-F), providing a mechanistic basis for the observed elevation in K1039 acetylation. Domain truncation studies coupled with Co-IP experiments localized Sirt3's binding region to the DNA_POL_gammaA domain of Polγ (Figure 5G, H). Protein spatial structure prediction revealed close proximity between the DNA_POL_gammaA domain and the D257 residue (Figure 5I), suggesting that the D257A mutation induced conformational changes that impair Sirt3 binding. Building on our structural analyses, we employed molecular docking to evaluate how the D257A mutation influenced the Polγ-Sirt3 interface. Our simulations revealed that this mutation induced a marked conformational rearrangement in Polγ structure. While the primary binding site remained structurally intact, the mutation substantially reduced binding affinity between Polγ and Sirt3. These observations suggested that D257A allosterically altered the interfacial dynamics, compromising the Polγ-Sirt3 interaction through indirect structural effects rather than direct disruption of the binding motif (Figure S1). Subsequent Co-IP assays confirmed that the D257A mutation universally reduced Polγ-Sirt3 interaction, independent of the K1039 site's acetylation status (Figure 5J, K). Ch-IP experiments corroborated these findings that D257A diminished mtDNA binding in Polγ-WT and K1039R, while the K1039Q mutant retained normal mtDNA association (Figure 5L). Collectively, these results establish a model wherein D257A disrupted Polγ-Sirt3 interaction, leading to hyperacetylation at K1039 (Figure 5M).

These findings highlight a critical regulatory axis linking functional integrity of Polγ, Sirt3-mediated deacetylation, and mtDNA maintenance. By using *POLG*^D257A/D257A^ premature aging mice, demonstrating the broad applicability of this novel regulatory mechanism across multiple biological systems, providing critical insights into the molecular basis of age-related functional decline.

### Hyper-acetylation at K1039 drives senescence via mitochondrial and functional deficits

**Figure 6:**
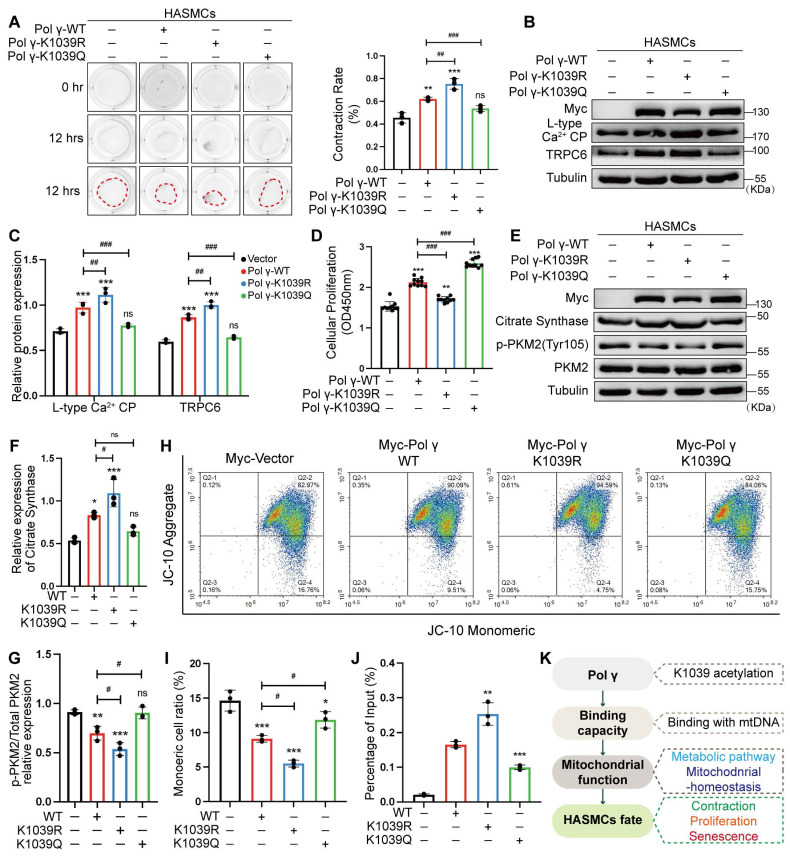
** Acetylated Polγ regulates HASMC function and mitochondrial homeostasis via mtDNA binding.** (A) Evaluate the effect of different acetylated Polγ in contractive ability by collagen gel contraction experiment. The percentage of gel surface area contraction (%) was calculated using the formula: [(0h area - 12h area) / 0h area] × 100%. ***p < .001, **p < .01, ^###^p < .001,^ ##^p < .01, ns p>0.05; (B-C) Transfect the HASMCs with various acetylated Polγ and compare the expression of L-type Ca^2+^ CP and TRPC6. The expression of Tubulin was used as a loading control. ***p < .001, ^###^p < .001,^ ##^p < .01, ns p>0.05; (D) Compare the proliferation alteration of HASMCs after transfecting various acetylated Polγ by CCK-8 assay. ***p < .001, **p < .01, ^###^p < .001; (E-G) Conduct WB analysis and quantification analysis to evaluate protein expression (citrate synthase, p-PKM2, PKM2, Tubulin) in HASMC which were transfected with different acetylated Polγ. The expression of Tubulin was used as a loading control for citrate synthase, and PKM2 was used as a loading control for p-PKM2. ***p < .001, ^#^p < .05, ns p>0.05; (H-I) The MMP alteration under various acetylated Polγ conditions by JC-10 assay. Compare the difference of monomeric cells ratio (green) in every subgroup. ***p < .001, *p < .05, ^#^p < .05; (J) The binding degree between different acetylated Polγ and mtDNA by Ch-IP assay. ***p < .001, **p < .01; (K) Schematic diagram. All data were expressed as the mean ± SD of triplicate experiments. In each independent experiment, the sample size for each group of experiments is 3.

To investigate the functional impact of Polγ acetylation at K1039, we transfected HASMCs with wild type Polγ (Polγ-WT), acetylation deficiency mimetic Polγ-K1039R or hyper acetylation mimetic Polγ-K1039Q. Overexpression of Polγ-WT enhanced HASMCs contractility, an effect further amplified by the K1039R mutation. In contrast, Polγ-K1039Q failed to augment contractility compared to the vector control (Figure 6A). WB revealed that Polγ-WT upregulated contractile markers LCP and TRPC6, with Polγ-K1039R exhibiting stronger induction, while Polγ-K1039Q showed no effect (Figure 6B, C). We found that Polγ promoted cell proliferation, intriguingly, Polγ-K1039Q also induced hyperproliferation, whereas Polγ-K1039R only modestly increased proliferation (Figure 6D). Metabolically, Polγ-WT elevated CS expression and suppressed PKM2 phosphorylation, promoting oxidative phosphorylation. These effects were enhanced by Polγ-K1039R but absent in Polγ-K1039Q-transfected cells (Figure 6E-G). In terms of mitochondrial homeostasis, we found that the MMP and functional integrity were robustly enhanced by Polγ-WT and further augmented by Polγ-K1039R, but only marginally improved by Polγ-K1039Q (Figure 6H, I). To assess binding capacity of Polγ, we performed Ch-IP assays to quantitatively measure the binding affinity between Polγ and mtDNA, which allowed us to establish a robust correlation between Polγ-mtDNA interaction dynamics. Ch-IP assays confirmed that Polγ-K1039R strengthened Polγ-mtDNA binding, while Polγ-K1039Q diminished this interaction (Figure 6J). To elucidate mechanistic insight into how K1039 acetylation regulates the mtDNA-binding capacity of Polγ, we performed structural predictions using AlphaFold. We chose and analyzed five independent models consistently revealed that K1039 acetylation induced significant substantial conformational rearrangements in Polγ, characterized by a transition from a compact to a more relaxed structural state. This acetylation-dependent structural relaxation likely impairs the precise spatial coordination required for effective mtDNA binding (Figure S2).

Herein, we propose a Polγ-mtDNA binding capacity-mitochondrial function-cellular function and fate regulatory axis. This axis delineates how Polγ modulates mitochondrial homeostasis via its acetylation, thereby dictating cellular phenotypes such as contraction or senescence (Figure 6K). Collectively, deacetylated Polγ could ensure the binding of Polγ to mtDNA, sustain mitochondrial oxidative metabolism, preserve the contractile phenotype of HASMCs, and prevent from senescence.

### K1039 deacetylation is essential for Polγ in maintaining mitochondrial homeostasis and HASMC function

**Figure 7:**
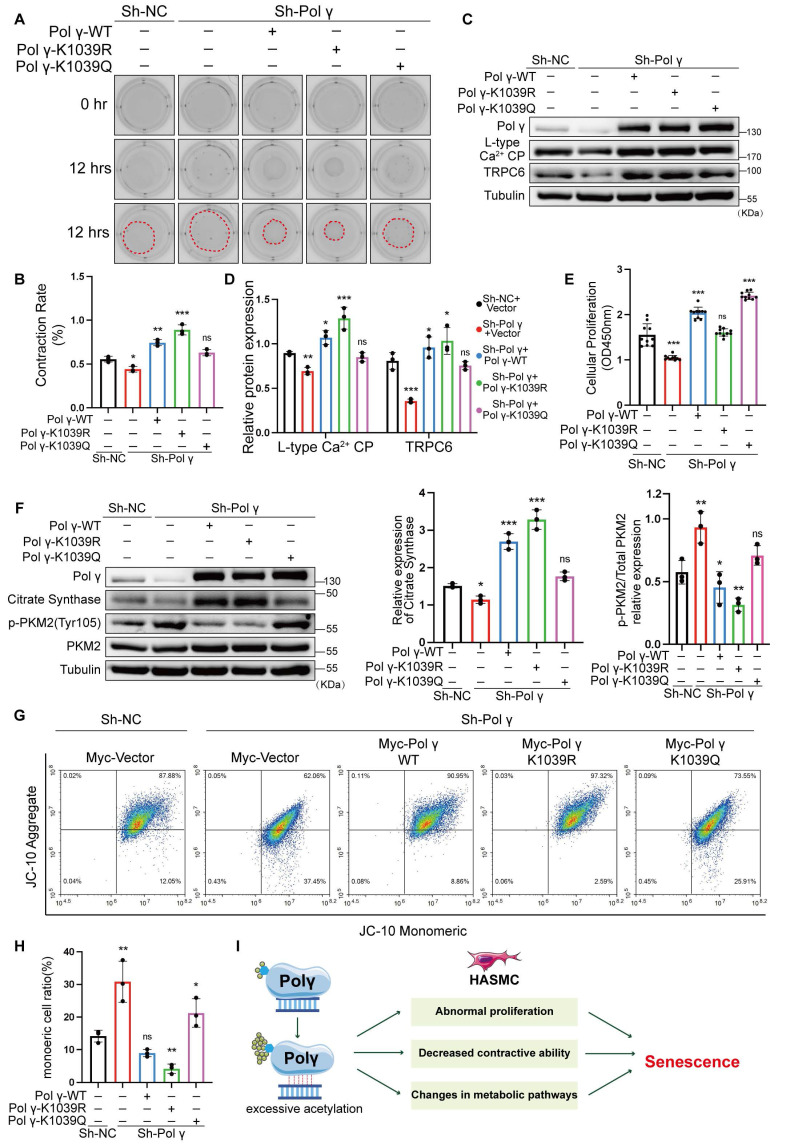
** K1039 acetylation status governs Polγ's role in metabolic and functional rescue.** (A-B) Transfect Sh-Polγ-HASMCs with various acetylated Polγ condition plasmid, evaluating the contractive ability by collagen gel contraction experiment. The percentage of gel surface area contraction (%) was calculated using the formula: [(0h area - 12h area) / 0h area] × 100%. ***p < .001, **p < .01, *p < .01, ns p>0.05; (C-D) Knock-down the expression of Polγ in HASMCs and rescue with various acetylated Polγ and compare the expression of L-type Ca^2+^ CP and TRPC6. The expression of Tubulin was used as a loading control. ***p < .001, **p < .01, *p < .01, ns p>0.05; (E) Evaluate the rescue effect of different acetylated Polγ in regulating the proliferation effect by CCK-8 assay. The results are defined as the absorbance in 450nm. ***p < .001, ns p>0.05; (F) Conduct WB analysis and quantification analysis to evaluate protein expression (citrate synthase, p-PKM2, PKM2, Tubulin) in Sh-Polγ-HASMC which were transfected with different acetylated Polγ plasmids for 48 hours. The expression of Tubulin was used as a loading control for citrate synthase, and PKM2 was used as a loading control for p-PKM2. ***p < .001, **p < .01, *p < .01, ns p>0.05; (G-H) The JC-10 assay to evaluate rescue effect of different acetylated Polγ in regulating MMP. Compare the difference of monomeric cells ratio (green) in every subgroup. **p < .01, *p < .01, ns p>0.05; (I). Schematic diagram. All data were expressed as the mean ± SD of triplicate experiments. In each independent experiment, the sample size for each group of experiments is 3.

To validate the causal role of K1039 acetylation in maintaining the HASMCs function and mitochondrial homeostasis, we performed rescue experiments in Polγ-knockdown (Sh-Polγ) HASMCs cell line. Polγ deficiency impaired contractility, which was fully restored by Polγ-WT and maximally rescued by Polγ-K1039R, while Polγ-K1039Q exhibited negligible rescue which was akin to negative controls (NC) (Figure 7A, B). Similarly, WB results showed that Polγ-K1039Q failed to upregulate expression of LCP and TRPC6, while Polγ-WT and Polγ-K1039R robustly increased their expression (Figure 7C, D). Polγ deficiency obviously decreased the proliferation of HASMCs and overexpression of Polγ restored fully its proliferation. Strikingly, Polγ-K1039Q exacerbated hyperproliferation in Sh-Polγ HASMCs, whereas Polγ-K1039R normalized proliferation to NC levels (Figure 7E). Metabolic profiling showed that Polγ deficiency reduced CS expression and elevated p-PKM2 level, indicative of glycolytic dominance. In rescue experiments, Polγ-WT and Polγ-K1039R restored CS levels and suppressed p-PKM2, but Polγ-K1039Q had minimal effect (Figure 7F). These results indicated it was deacetylated Polγ that ensured metabolic homeostasis which was indicative of aerobic respiration dominance. Except for comprehensive metabolic pathway analysis, mitochondrial membrane potential dynamics were quantitatively monitored to evaluate role of acetylated Polγ in maintain mitochondrial homeostasis and determinate mitochondrial fate. JC-10 assays mirrored these trends that Polγ deficiency markedly reduced MMP, thereby severely compromising mitochondrial integrity and homeostatic maintenance as previous results. But Sh-Polγ-induced mitochondrial dysfunction was fully rescued by Polγ-WT and Polγ-K1039R, especially the Polγ-K1039R had maximally rescue effect, while Polγ-K1039Q provided partial recovery (Figure 7G, H).

These findings demonstrated K1039 deacetylation was essential for sustaining Polγ's mtDNA-binding capacity. Elevated acetylation at the K1039 residue inversely correlates with Polγ binding capacity with mtDNA and molecular function. Hyperacetylation at K1039 disrupted mtDNA binding, impaired oxidative metabolism, and driven HASMCs toward a proliferative, senescence-prone phenotype (Figure 7I).

## Discussion

Here, our study established a conceptual advance in Polγ functional regulation by demonstrating that acetylation fundamentally modulated its mtDNA binding capacity. We demonstrate that Polγ undergoes GCN5/Sirt3-mediated acetylation at K1039, which was markedly enhanced during cellular senescence. Acetylation of K1039 attenuates Polγ binding to mtDNA, impairing mitochondrial function and driving both functional decline and senescence in HASMCs. Strikingly, hyperacetylation of K1039 or introduction of D257A mutation reduced the binding between Polγ and mtDNA, compromised mitochondrial integrity, and ultimately precipitated HASMC senescence through loss of contractile capacity.

As a critical mitochondrial regulator, POLG governs cellular fate by maintaining mitochondrial functional homeostasis and ATP balance^19^-^21^. This is particularly vital in high-energy-demand tissues such as muscle and brain, consequently, POLG deficiency frequently manifest as myopathies and neuropathies, such as activation of neurotoxic astrocytes and peripheral neuropathy^21^-^24^. In 2001, pioneering work by Van Goethem *et al*. identified four *POLG* mutations in patients with progressive external ophthalmoplegia (PEO), establishing a causal link between Polγ deficiency and disease 25. Subsequently, mutations in *POLG* were discovered in patients with ataxia 26-28. Furthermore, *POLG* mutations were confirmed as the cause of Alpers-Huttenlocher syndrome (AHS), marking a seminal breakthrough in understanding the molecular basis of this *POLG*-related disorder^29^, ^30^. However, it is particularly noteworthy that limited evidence from clinical disorders or mechanistic studies implicated reduced Polγ expression as the primary cause of mitochondrial functional collapse. Instead, various large-scale clinical analyses have confirmed the pathogenic mechanism predominantly arisen from mutations that impair catalytic activities of Polγ 4. Consistent with this, the D257A mutation animal model employed in this study similarly exhibited unchanged Polγ expression, but displayed a 5-fold increase in mtDNA defect 15, 16, 31. Although these mutations took place across various domains and involve multiple amino acid residues, they uniformly resulted in mtDNA defects and depletion. This feature strongly suggested that all pathogenic POLG mutations ultimately impair its polymerase or exonuclease activities, leading to catastrophic mtDNA failure. This phenomenon supported our hypothesis that Polγ acted as a central integrator and signal transducer for diverse mitochondrial damage signals. While some studies suggest extramitochondrial functions for Polγ^32^, ^33^, but we believed the primary pathway by which Polγ deficiency altered cellular fate was through mtDNA dysregulation. Consequently, these findings strongly implied the existence of an underlying regulatory mechanism capable of precisely coordinating the POLG-mtDNA interaction.

Our findings position K1039 acetylation as a central regulator of HASMC homeostasis and senescence. While prior studies linked Polγ deficiency or mutation to aging-associated pathologies^4^, ^5^, ^34^, we identify elevated K1039 acetylation as a unifying molecular signature across diverse senescence models, including *POLG*^D257A/D257A^ mice. Mechanistically, the D257A mutation disrupted Sirt3-Polγ interaction, exacerbating K1039 acetylation and diminishing mtDNA binding—a prerequisite for Polγ-mediated transcription of respiratory chain components^4^, ^35^. We propose that diverse damage signals converge on K1039 hyperacetylation to modulate Polγ activity indirectly, rather than directly targeting Polγ-mtDNA binding. This paradigm reconciles previous observations that both Polγ depletion and catalytic impairment accelerate senescence^34^, ^36^. positioning acetylation as a tunable regulator of mitochondrial genome maintenance. Notably, K1039 acetylation precipitated metabolic reprogramming from oxidative phosphorylation to glycolysis which is a hallmark of senescent cells. By disrupting mtDNA replication and respiratory complex assembly^37^, hyperacetylated Polγ forces HASMCs into an energetically inefficient glycolytic state, culminating in mitochondrial collapse. Concomitant loss of contractile function and aberrant proliferation mirrored phenotypic switching observed in vascular aging^38^, ^39^. Our data align with the Hayflick limit paradigm^40^, ^41^, wherein replicative exhaustion triggers senescence, but extend this concept by implicating acetylation-driven mitochondrial failure as an accelerator of proliferative arrest.

Our current findings demonstrate that K1039 acetylation significantly impairs Polγ's binding affinity for mtDNA. Given that the D257A mutation similarly weakens this interaction, we hypothesize that K1039 acetylation may modulate the exonuclease activity of Polγ. This conjecture is supported by our previous work showing that the exonuclease-deficient D257A mutant triggered excessive p53 recruitment to mitochondria, where it interacted with Polγ to help maintain mtDNA quality control. Collectively, our data support a model wherein impaired Polγ-mtDNA binding, while not affecting intrinsic polymerase activity, disrupts mtDNA quality control in a binding-dependent manner and ultimately leads to mitochondrial dysfunction and HASMC cellular impairment.

Furthermore, while several studies have reported decreased Polγ expression in diverse pathological conditions 10, 33. We noted that these conditions typically involve multifactorial pathological insults processes, within these complex processes, Polγ dysregulation likely represented one component influenced by up-stream signaling events. This suggested that cellular fate under such pathological conditions wasn't solely determined by the Polγ pathway. In contrast to these correlative observations, our study employed definitive rescue experiments. Specifically, we performed Polγ knockdown followed by transfecting with various Polγ plasmids (K1039K, K1039R and K1039Q), mimicking distinct acetylation states, directly established the acetylation status of Polγ as the primary triggering event for mitochondrial dysfunction and cellular senescence. Technical limitations merit consideration. Structural occlusion of K1039 within Polγ's folded conformation precluded development of site-specific acetyl antibodies. Residual acetylation in K1039-mutant Polγ suggests auxiliary modification sites, though the profound functional impact of K1039R underscores its primacy in regulating senescence. Future studies employing Polγ K1039R knock-in models will clarify the spatiotemporal dynamics of this modification in aging.

In conclusion, we delineate a GCN5/Sirt3-Polγ axis wherein K1039 acetylation serves as a molecular switch governing mitochondrial fidelity and cellular aging. By linking post-translational regulation of Polγ to metabolic homeostasis and phenotypic plasticity of HASMCs, our work unveils novel therapeutic opportunities for age-related vascular pathologies. This mechanistic bridge between mtDNA maintenance and protein acetylation networks establishes Polγ as a linchpin target for interventions against degenerative aging.

## Supplementary Material

Supplementary figures.

## Data Availability

The original mass spectrometry data reported in this paper will be shared by the Prof. Yingxian Sun upon request. Any additional information required to reanalyze the data reported in this paper is available from Prof. Yingxian Sun (yxsun@cmu.edu.cn).
