# Supplementary material for: DNA Polymerase Gamma Acetylation Governs Mitochondrial Homeostasis and Vascular Cell Senescence
Source: Int J Biol Sci. 2026 Feb 4;22(5):2435–51. doi: 10.7150/ijbs.122298 (PMC12965149; doi:10.7150/ijbs.122298)
Supplement: Supplementary file 1 — Supplementary figures. [file ijbsv22p2435s1.pdf]

Figure S1. D257A mutation reduced binding between Polγ and Sirt3 by molecular docking.

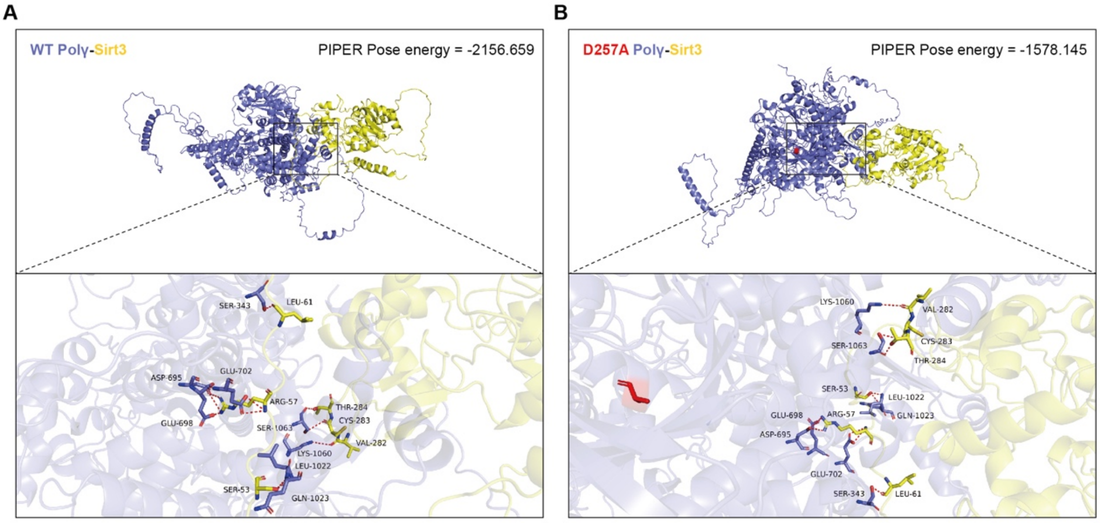

D257A mutation reduced binding between Polγ and Sirt3 by molecular docking. (A) The predicted structural model of the wild-type Polγ- Sirt3 complex, with Polγ depicted in blue and Sirt3 in yellow. The interface analysis predicts potential interfacial residues mediating the interaction between the two proteins. (B) The predicted structural model and potential interfacial residues of the D257A mutated Polγ- Sirt3 complex, with Polγ depicted in blue (D257 site in red) and Sirt3 in yellow.

Figure S2. K1039 acetylation altered the spatial structure of Polγ.

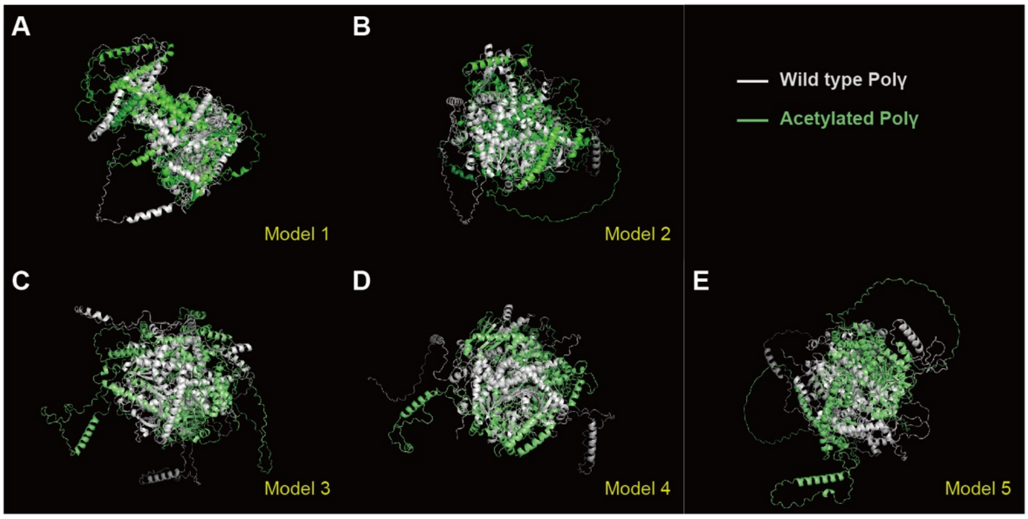

K1039 acetylation altered the spatial structure of Polγ. (A-E) AlphaFold3 was employed to predict the structures of both wild-type Polγ and K1039-acetylated Polγ (acetyl group in K1039 site). Five representative structural models were generated for each form.
